# Stain Susceptibility of 3D-Printed Nanohybrid Composite Restorative Material and the Efficacy of Different Stain Removal Techniques: An In Vitro Study

**DOI:** 10.3390/ma14195621

**Published:** 2021-09-27

**Authors:** Nawal Alharbi, Amal Alharbi, Reham Osman

**Affiliations:** 1 Department of Prosthetic Dental Sciences, College of Dentistry, King Saud University, Riyadh 12372, Saudi Arabia; 2 Operative Dentistry, AlNakheel Medical Centre, Riyadh 11564, Saudi Arabia; amalmarshed@gmail.com; 3 Prosthodontics Department, Faculty of Dentistry, Cairo University, Cairo 11311, Egypt; rehambosman@gmail.com

**Keywords:** 3D-printing, additive manufacturing, SLA, restorations, CAD/CAM

## Abstract

Recent burgeoning development in material science has introduced a 3D-printable, nanohybrid composite resin restorative material. However, its performance has not yet been investigated. This study evaluates the stain susceptibility and efficacy of different stain removal techniques. A total of 120 labial veneers were fabricated using milling (*n* = 60) and SLA 3D-printing (*n* = 60). Based on the immersion media: coffee, tea and artificial saliva, each group was divided into three sub-groups (*n* = 20). Stain susceptibility was evaluated by calculating color difference (∆E_00_) at 12 and 24 days using a spectrophotometer against black and white backgrounds. Collected data were analyzed with ANOVA and Tukey’s post hoc test (*p* < 0.05). A significant interaction effect was found between the staining mediums and fabrication methods in both black and white backgrounds (*p* < 0.001). 3D-printed restorations showed significantly higher stain susceptibility than milled restorations (*p* < 0.001). Prolonged immersion time increased the color difference in both groups. In-office bleaching was more effective in stain removal in both 3D-printed and milled restoration groups. The susceptibility of the presented novel 3D-printed restorative material to color changes in different immersion mediums was clinically not-acceptable. The clinicians might expect the need to replace the restoration after 1–2 years and thus, recommendation for the use of such a material as a permanent restoration cannot be made but rather as a long-term temporary restoration.

## 1. Introduction

Rapid advancements of digital tools and biomaterials has resulted in the development of an increased number of diagnostic tools, manufacturing technologies and material alternatives to conventional resin restorative techniques [1,2,3]. The additive manufacturing technique (AM) is the process of building the object by stacking one layer on top of another until the desired object is completed [4]. Dental restorations, resin dentures, study models and esthetic mock-up have all been successfully fabricated using AM technology, and the technique is not considered novel anymore [4,5]. Recent developments in the field of biomaterials have resulted in the introduction of a novel printed nanohybrid composite resin material that has been recommended for use as a definitive restorative material.

One of the main goals of using either a permanent or a provisional restorative material is to offer the patients an esthetic substitute to lost tooth structure. However, the complex harsh intra-oral environment may affect the physical and mechanical properties of the used restorative material. Color stability of restorative materials is known to be influenced by different types of stains to which the material is exposed, composition and surface roughness of the restorative material, as well as the frequency and duration of exposure time. Frequent exposure to coloring beverages is reported to significantly influence the color stability of the conventional resin restorative materials. Another factor that may be influential on the color stability of any restorative material is the manufacturing technique that is used. The stair-stepping phenomena which is commonly observed with 3D-printing technology, especially on curved surfaces, may be of significant importance when considering the color stability and consequently the esthetics provided by 3D- printed resin materials used for anterior esthetic restorations [4,6]. Furthermore, the post-polymerization procedure employed as a part of the AM technique influences the final structure of the material and hence can have a direct effect on the color stability of the printed material [7].

Recent studies have shown that the color stability of the 3D-printed resin restorative materials is significantly lower when compared to that of the milled CAD/CAM materials [8,9,10]. On the other hand, water sorption is higher and also differs among the different available printed materials [9]. A novel nano-composite 3D-printed material has been introduced in the market and recommended for use as a definitive long-term restorative material. However, it is of utmost importance that the mechanical and physical properties of such a material be tested to enable evidence-based recommendations and not solely relying on marketing information.

Several methods have been proposed to remove the stains from resin restorative materials [11,12,13,14]. In-office bleaching has been shown to be a more conservative method in stain removal when compared to surface polishing. The effectiveness of the bleaching technique on stain removal is dependent on the type of stain and the composition of the used material [14]. Whether the same applies to newly introduced printed resin restorative material or not is yet to be investigated.

Therefore, the aim of this in vitro study was to evaluate stain susceptibility and the color stability of the novel introduced 3D-printed, nanohybrid composite resin, permanent restorative material, and furthermore, to evaluate and compare the efficacy of in-office bleaching and surface polishing techniques on stain removal.

## 2. Materials and Methods

An incisal-wrap labial veneer of an upper central incisor was digitally designed using 3-Shape Dental System^TM^ CAD solution version 2015. The thickness of the veneer was 1 mm, as measured at the mid-labial surface. The digital design file was exported in a Standard Tessellation Language (STL) format and used to fabricate the test specimens (*n* = 120) using both milling and 3D-printing techniques. Sixty veneers were 3D-printed using a SLA-printer (DFAB; DWS; Thiene, Italy) with a nano-composite resin material (Shade A2; Irix Max; DWS; Thiene, Italy) [15]. Layer thickness was 0.05 mm and maximum laser speed was 5000 mm/s at a printing angle of 180°, where the layers were stacked along the height of the specimen [6,16]. All specimens were cleaned with 95% ethanol for 1 min and post-processed in an ultraviolet light-curing unit (Dcure; DWS; Thiene, Italy) for 5 min following the manufacturer’s instructions. In the milling group, 60 veneers were milled from Cerasmart composite resin material (Cerasmart^®^ shade A2; GC, Tokyo, Japan) using a 5-axis milling machine (Ceramill motion 2; Amann Girrbach, Koblach, Austria). All specimens were visually inspected for manufacturing defects and were subsequently polished using Soflex^®^ discs from medium to superfine (SofLEX; 3M; USA) by one trained examiner (A.A.).

All the specimens in both groups (*n* = 60 milled and *n* = 60 3D-printed) were cleaned using distilled water in an ultrasonic cleaner. Specimens in each group were randomly divided into 3 subgroups (*n* = 20) depending on the immersion medium: artificial saliva (Glandosane^®^, Helvepharm AG), black tea (Lipton^®^) and coffee (Arpeggio; Nespresso^®^; Switzerland). In each subgroup, the specimens were immersed for 12 and 24 days and stored in an incubator at 37 °C. Artificial saliva was used as delivered, tea was prepared by dissolving a tea bag in 100 mL of boiling water and coffee was prepared as an espresso coffee using an Arpeggio coffee capsule.

**Figure 1:**
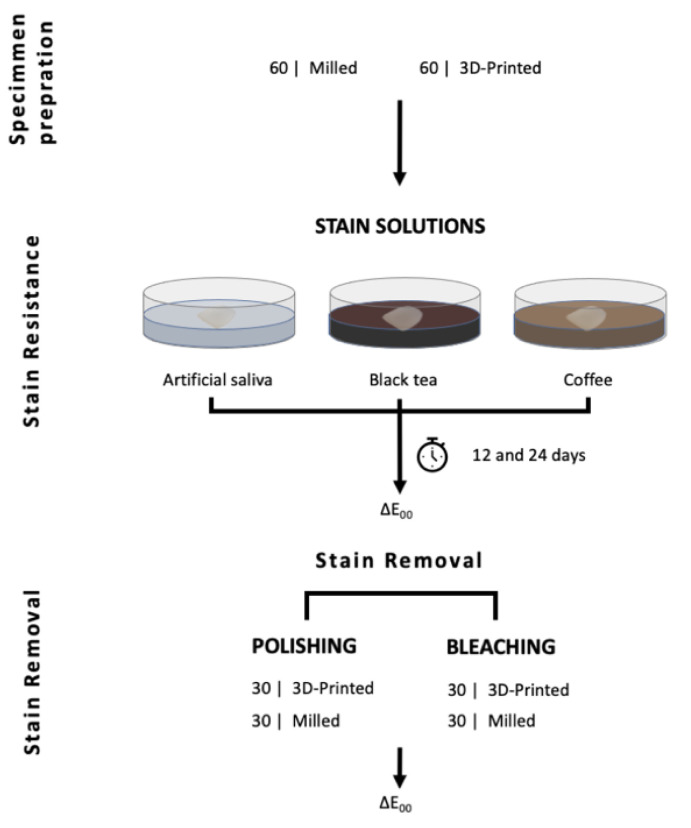
Experiment flowchart.

The immersing mediums were refreshed every week to avoid bacterial or yeast contamination. Figure 1 shows a complete flowchart of the experimental setup.

### 2.1. Color Measurement

Color stability/stain susceptibility was evaluated by calculating the color difference (∆E_00_) using the formula developed by the International Commission on Illumination (CIEDE2000) against both a black and a white background. An average of three measurements was obtained from each specimen at each timepoint using a calibrated spectrophotometer clinical device (VITA Easyshade^®^V; VITA Zahnfabrik, Germany) [17,18]. All measurements were taken at the mid-labial surface, and the device tip was positioned 3 mm apical to the incisal edge. Measurements were taken at baseline 24 h after specimens’ preparation, and at 12 and 24 days after immersion in different staining mediums against black and white backgrounds. The color changes were calculated according to the CIEDE2000 (∆E_00_) formula [9,19,20]:

(1) $$∆E00=\sqrt{{\left(\frac{∆L}{K_{L}S_{L}}\right)}^{2}+{\left(\frac{∆C}{K_{C}S_{C}}\right)}^{2}+{\left(\frac{∆H}{K_{H}S_{H}}\right)}^{2}+R_{T}\left(\frac{∆C}{K_{C}S_{C}}\right)\left(\frac{∆H}{K_{H}S_{H}}\right)}$$

Values of ∆E_00_ (perceptibility threshold) ≤ 0.8 denote that the color difference is not perceptible/noticeable by the human eye. Values of ∆E_00_ ≤ 1.8 are perceptible but are still clinically acceptable. Values of ∆E_00_ ≤ 3.6 are considered moderately unacceptable, ∆E_00_ ≤ 5.4 are considered clearly unacceptable and ∆E_00_ > 5.4 are extremely unacceptable [20]. Bleaching effectiveness were considered excellent effectiveness if ∆E_00_ > 5.4, very good effectiveness if ∆E_00_ ≤ 5.4, good effectiveness if ∆E_00_ ≤ 3.6 and moderately effective if ∆E_00_ ≤ 1.8 [20].

### 2.2. Stain Removal

The stained specimens in each immersion subgroup were randomly divided to receive either of two surface treatments: in-office bleaching group (*n* = 30) or surface polishing (*n* = 30). The in-office bleaching was performed using 40% hydrogen peroxide (Opalescence^®^ Boost PF 40%; Ultradent products, Inc., UT) for one hour [14]. Bleaching coat of ~1 mm thickness was refreshed after 30 min, rinsed with water for 30 s and then was dried with tissue paper. Color changes were measured following the above-described technique. In the polishing group (*n* = 30), the labial surface of the specimens was polished for 60–80 s using a sequenced grit roughness Soflex^®^ disc from medium to superfine (3M, USA) following a previously reported polishing protocol [21].

### 2.3. Statistical Analysis

The results were analyzed using SPSS statistics (IBM SPSS statistics for MAC, v28; IBM Corp). The data were checked for normality of distribution and equivalence of variance using the Schapiro–Wilk test. Analysis of variance (ANOVA) was used to evaluate the effect of the material and staining medium on the color difference, and the efficacy of stain removal methods was evaluated with Tukey’s post hoc test. Statistical significance was set at *p* < 0.05. The null hypothesis was that there is no difference between printed and milled material in susceptibility to stains. The second hypothesis was that there is no difference in efficacy of stain removal between polishing and in-office bleaching techniques.

## 3. Results

**Figure 2:**
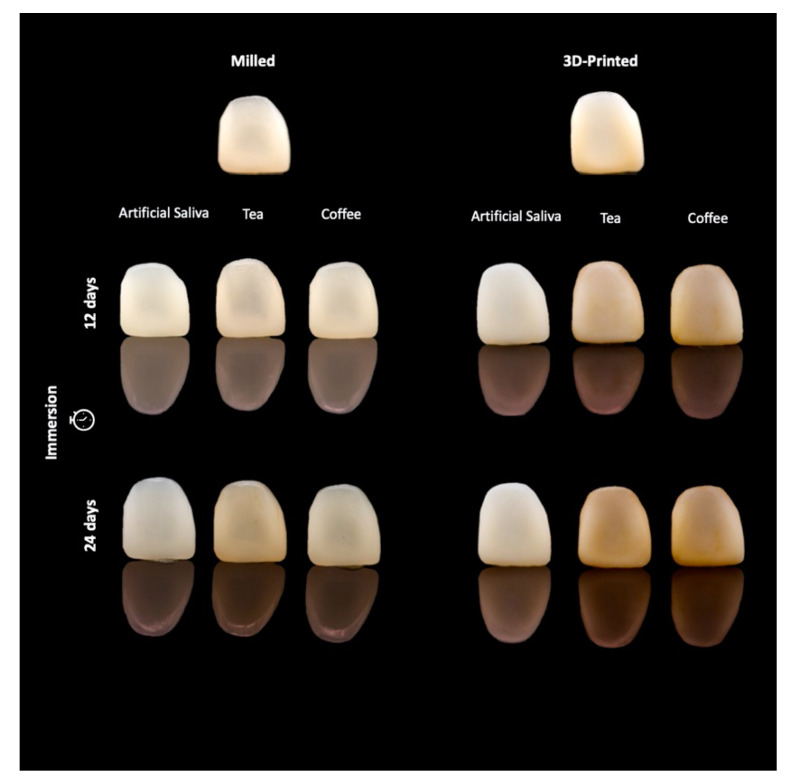
Photograph of ∆E_00_ of stained milled and 3D-printed specimens after 12 and 24 days of immersion in staining mediums.

Mean and standard deviation of the color difference values (∆E_00_) among different staining mediums are presented in Table 1. Two-way ANOVA revealed a statistically significant interaction between the effect of staining mediums and fabrication methods in both black and white backgrounds, *p* < 0.001 (Table 2). Simple main effects analysis showed that ∆E_00_ was significantly higher in printed restorations compared to milled restorations in all test mediums in both black and white backgrounds (*p* < 0.001). Color changes observed after immersion in coffee and tea staining mediums were clinically unacceptable (Figure 2).

ANOVA with repeated measurements revealed a significant interaction effect for both fabrication time and staining mediums (F = 247.73, *p* < 0.001). Prolonged immersion in tea and coffee for 24 days resulted in significantly higher ∆E_00_ in printed restorations (*p* = 0.001) for both black and white backgrounds. Prolonged immersion in artificial saliva showed no significant difference in ∆E_00_ between milled (F = 0.01, *p* = 0.938) and printed restorations (F = 3.51, *p* = 0.064). A similar observation was noticed with the white background for milled restorations (F = 0.41, *p* = 0.839), whereas for 3D-printed restorations, the values were F = 7.109, *p* = 0.009.

**Figure 3:**
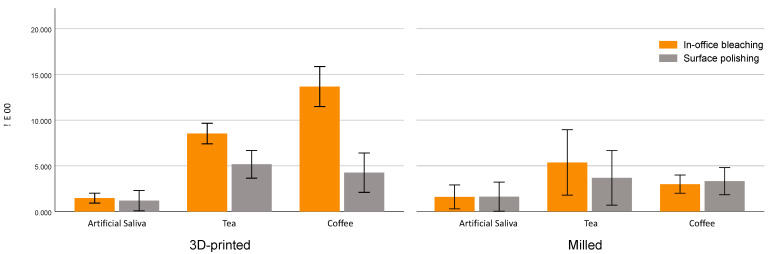
∆E_00_ of stained milled and 3D-printed specimens after in-office bleaching and polishing techniques after immersion in coffee, tea and artificial saliva.

**Figure 4:**
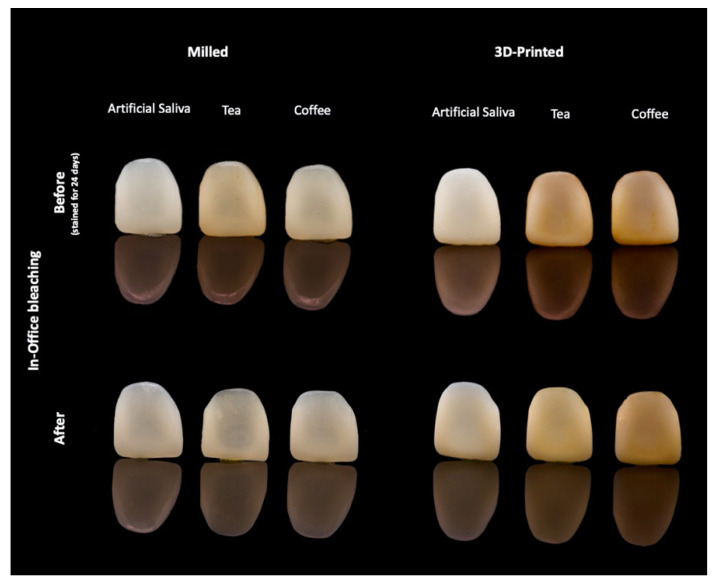
Photograph showing the effect of in-office bleaching in both 3D-printed and milled restoration groups.

The efficacy of in-office bleaching and surface polishing on stain removal is shown in Table 3 and Figure 3. One-way ANOVA revealed a significant interaction effect between fabrication methods, staining mediums and type of treatment (F = 71.39, *p* < 0.001). Pairwise comparison revealed that surface polishing resulted in lower ∆E_00_ values compared to the bleaching technique (F = 189, *p* < 0.001). Tukey’s post hoc test showed no statistically significant difference in ∆E_00_ in coffee-stained milled restorations (*p* = 0.443), and artificial saliva in milled (*p* = 0.945) and 3D-printed restorations (*p* = 0.524) between surface polishing and bleaching treatments. Bleaching revealed excellent effectiveness in stain removal for 3D-printed restorative material (∆E_00_ = 7.87) and good effectiveness with milled material (∆E_00_ = 3.33) [20]. Figure 4 shows the effect of in-office bleaching in stain removal for both 3D-printed and milled restorations.

## 4. Discussion

Based on the results of the present in vitro study, the null hypothesis that there would be no difference in color changes of 3D-printed and milled composite restorative material immersed in different staining mediums was rejected. The second hypothesis that there would be no difference between the efficacy of the bleaching and polishing techniques on stain removal was also rejected.

Acknowledging the fact that the color measurement is influenced by the surrounding conditions as well as the geometry and thickness of the tested specimens, the design of a labial veneer restoration was selected for this study to best simulate clinical conditions compared to standard disc-shaped specimens commonly used in other color detection studies [8,9,14,19,22,23,24]. Furthermore, when the 3D-printing technique is used to fabricate anterior restorations, stair-stepping phenomena is anticipated, especially with the characteristic curved contour of such restorations [3]. This phenomenon will influence surface roughness and subsequently the physical properties of the restoration [6]. All restorations were printed at a 180° build angle, which is shown to offer better dimensional accuracy and less surface roughness [4,6,16].

All measurements were taken against both black and white backgrounds to simulate light reflectance on several clinical conditions, CIV, CV, CII and CI [19,25]. The results revealed that the influence of the background was minimal, and differences in the results between white and black backgrounds after immersion in artificial saliva might be explained by the difference in the translucency of the milled and 3D-printed materials [19] (Table 1 and Table 2). Several methods are available and are used to detect color difference in dental restorations, among which, the clinical spectrophotometer is a calibrated and well-established device for quantitative color measurement [17,18]. Color difference was calculated based on the formula CIEDE2000 (ΔE_00_), which is recommended for better representation of human perceptions of color difference when compared to the CIELAB formula [19,20].

Clinically, dental restorations are expected to be exposed to various staining beverages. The choice of beverages used in this study was based on their frequent use in real life among different cultures, their availability, as well as the fact that their staining ability is widely studied in the literature. All specimens were immersed for 12 and 24 days in staining mediums, time periods which are equivalent to one and two years of intraoral exposure, respectively.

The findings of our study are in agreement with previous studies [23,25,26]. Coffee showed more color difference compared to tea and artificial saliva in the 3D-printed material at both time intervals. Tannin and chlorogenic acid in coffee have been shown to diffuse within the structure of the immersed material and consequently cause its discoloration. Further, the pH of coffee ranges from 4.9 to 5.2, a range of values which has been reported to accelerate the staining capacity of material [12,27,28] (Table 1, Figure 2). In this experiment, all tested materials were immersed in coffee and tea, and showed ΔE_00_ values higher than the acceptability threshold, which is reported to be ΔE_00_ ≤ 1.8 in the literature [20]. Therefore, the color changes which were observed in this study for both material groups upon immersion in tea and coffee are considered clinically unacceptable for permanent long-term use, and restoration replacement may be required after a time interval of 1 or 2 years. Patients who do not consume tea or coffee might still experience changes in the color of their restorations, as revealed by color changes of materials upon immersion in saliva, however the range of color change is considered to be clinically acceptable.

In accordance with previous studies on temporary printed restorative materials [8,9,29], the results of the current in vitro experiment revealed that the tested 3D-printed material exhibited higher stain susceptibility compared to the milled material (*p* < 001). Higher stain susceptibility of 3D-printed material may be attributed to the manufacturing technique, which results in multiple layers one stacked on top of the other. Possible incomplete polymerization at the layer interface and presence of microporosities and residual monomers can definitely contribute to increased discoloration potential of printed materials [9].

CAD/CAM blocks used for milled restorations are industrially polymerized at high pressure and temperature to optimize the polymerization process, which results in a more compact structure with improved mechanical and physical properties. The resin matrix composition in the tested milled CAD/CAM material is urethane dimethacrylate (UDMA), and this can explain the higher color stability observed in this group when compared to the 3D-printed material (Figure 2, Table 1). Though no information was provided or available regarding the material composition in 3D-printed material, we can speculate that the resin matrix of material is composed of BIS-GMA. BIS-GMA exhibits low viscosity, a quality which is needed for printable materials using any of the available vat photo-polymerized AM technologies to facilitate the manufacturing process and the flow of the material through the nozzle of the printer without any clogging during the printing process. It is thus of great importance that parallel to and in line with burgeoning advancements in the field of biomaterials, basic information about the novel marketed materials should be provided to enable better evidence-based decisions by the dental practitioners. Furthermore, improving the material structure and filler content are factors that are yet to be evaluated and tested by the manufacturer in close collaboration with clinical research centers to improve the physical and mechanical properties of the newly introduced 3D-printed materials.

The difference in color stability between the tested materials can also be related to the difference in the materials’ composition and the microstructure of the resin material involving the type of resin matrix and the filler content of the material. Susceptibility to color change may result from sorption of the stains into the organic matrix. The bisphenol A glycol dimethacrylate (BIS-GMA) matrix is known to have a high water-absorption rate. On the other hand, absence of the hydroxyl side group in urethane dimethacrylate (UDMA) results in a less hydrophilic, more viscous matrix, which results in increased color stability of the milled material and is widely used in hybrid composite resin restorative materials [30,31]. The mechanism of coffee staining depends on the adsorption and sorption of the staining medium within the organic matrix of the material. Thus, the higher susceptibility of milled restoration with UDMA matrix to tea stains can be explained by the fact that the tea is adsorbed to the surface whereas coffee is not able to diffuse to the matrix, hence the increased susceptibility to color changes with tea, whereas coffee resulted in more staining and color change of the printed restorative material, probably owing to the BIS-GMA matrix content of the material. However, such an explanation could not be critically ascertained as the exact filler and matrix content of novel 3D-printed material was not disclosed by the manufacturer.

Oxidation of unreacted residual monomers in the matrix is another factor that may contribute to color changes of material, even without subjecting the material to colorant stains. Polymerization rate and the post-polymerization process have been reported to influence the accuracy of the printed parts [7]. This highlights the importance of proper execution of the post-processing step of any printed restorative material as per manufacturers’ recommendations to prolong the survival of restorative material.

Various methods are employed to remove the stains from teeth and dental restorative materials [13,25,27,32]. Bleaching is shown to be a more conservative and effective method to remove the stains [14]. The peroxide in the bleaching agent will decompose into free radicals that will diffuse into the material and breakdown the pigmentation molecule, and therefore remove or decrease stains [12,32]. On the other hand, surface polishing is based on surface abrasion of the treated surface of the material [22]. The results of the current study showed that in-office bleaching was more effective in both material groups in stain removal than surface polishing (ΔE_00_ > 1.8), which may indicate that the stain was incorporated in the matrix and not only adsorbed on the surface of the specimens (Figure 3 and Figure 4).

The current study is the first to test the stain susceptibility/color stability and efficacy of stain removal techniques in permanent 3D-printed resin material. Though this is an in vitro experimental design, the results provide clinicians with valuable information on the material response to stained beverages. The limitations of the presented in vitro study are associated with the limited number of available materials with similar composition for additional comparisons. Only coffee and tea staining mediums were selected due to their popularity. Whether the materials need replacement after 1–2 years needs to be verified clinically. The lack of detailed information about the composition of the presented material limits our conclusion of the study. Further research should explore the effect of different printing techniques, various materials with different compositions and staining mediums on the color stability of novel introduced 3D-printed materials. The exact degree of conversion of the printed material is still to be explored in future studies, as well as the influence of post-polymerization procedures.

## 5. Conclusions

The color changes of the presented novel 3D-printed restorative material were very high, and thus at present, with the current composition, recommendation for the use of the material as a permanent restoration cannot be made. The color changes in both milled and 3D-printed material indicate the need for replacement of the material after 1–2 years, and accordingly, the material can be recommended for use as a long-term temporary restoration. The efficacy of stain removal was higher with an in-office bleaching technique compared to surface polishing.

## Figures and Tables

**Table 1:** Mean (SD) of ∆E_00_ of 3D-printed and milled specimens after immersion in artificial saliva, tea and coffee for 12 and 24 days.

		Mean ∆E_00_ (±SD)			
	Time	Immersion 12 Days		Immersion 24 Days	
Fabrication	Background	Black	White	Black	White
	Stain				
3D-Printing	Artificial saliva	0.64 (±26)	0.63 (±0.26)	1.15 (±0.26)	1.34 (±0.25)
	Tea	9.61 (±0.68)	9.36 (±0.84)	14.83 (±0.84)	14.79 (±1.07)
	Coffee	19.65 (±1.35)	19.66 (±1.11)	32.47 (±1.29)	32.68 (±1.51)
	Total	9.97 (±7.88)	9.88 (±7.88)	16.15 (±12.96)	16.27 (±12.99)
Milling	Artificial saliva	1.43 (±0.33)	1.53 (±0.34)	1.41 (±0.71)	1.48 (±0.74)
	Tea	3.83 (±1.33)	3.85 (±1.27)	7.80 (±1.78)	7.61 (±1.89)
	Coffee	3.95 (±1.02)	3.62 (±0.73)	5.42 (±0.71)	5.33 (±0.58)
	Total	3.07 (±0.52)	3.00 (±1.36)	4.88 (±2.90)	4.81 (±2.82)
Total	Artificial saliva	1.03 (±0.5)	1.08 (±0.54)	1.28 (±0.55)	1.41 (±0.55)
	Tea	6.72 (±3.11)	6.60 (±2.99)	11.31 (±3.82)	11.20 (±3.94)
	Coffee	11.80 (±8.04)	11.64 (±8.18)	18.95 (±13.73)	19.01 (±13.89)
	Total	6.52 (±6.63)	6.44 (±6.61)	10.51 (±10.93)	10.54 (±10.99)

**Table 2:** Results of two-way ANOVA, interaction effect in black and white background for 12 and 24 days.

	Source—Background	Type III Sum of Squares	df	Mean Square	F	Sig
12 days	Fabrication * Stain—Black	1378.51	2	689.25	786.23	<0.001
	Fabrication * Stain—White	1462.95	2	731.48	1022.98	<0.001
24 days	Fabrication * Stain—Black	3998.87	2	1999.44	1809.49	<0.001
	Fabrication * Stain—White	4052.27	2	2026.13	1524.09	<0.001

**Table 3:** Mean and SD of ∆E_00_ after subjecting stained specimens to in-office bleaching and surface polishing techniques.

Fabrication	Stain	Treatment	Mean ∆E_00_ (±SD)
3D-Printing	Artificial saliva	Bleaching	1.46 (±0.27)
		Polishing	1.19 (±0.56)
		Total	1.33 (±0.45)
	Tea	Bleaching	8.53 (±0.56)
		Polishing	5.17 (±0.76)
		Total	6.85 (±1.84)
	Coffee	Bleaching	13.67 (±1.10)
		Polishing	4.25 (±1.08)
		Total	8.96 (±4.95)
	Total	Bleaching	7.89 (±5.14)
		Polishing	3.53 (±1.90)
		Total	5.71 (±4.42)
Milling	Artificial saliva	Bleaching	1.60 (±0.65)
		Polishing	1.63 (±0.80)
		Total	1.62 (±0.71)
	Tea	Bleaching	5.37 (±1.79)
		Polishing	3.69 (±1.50)
		Total	4.53 (±1.82)
	Coffee	Bleaching	3.00 (±0.50)
		Polishing	3.33 (±0.74)
		Total	3.17 (±0.64)
	Total	Bleaching	3.33 (±1.93)
		Polishing	2.88 (±1.38)
		Total	3.11 (±1.67)
Total	Artificial saliva	Bleaching	1.53 (±0.49)
		Polishing	1.41 (±0.71)
		Total	1.47 (±0.60)
	Tea	Bleaching	6.95 (±2.07)
		Polishing	4.43 (±1.38)
		Total	5.69 (±2.16)
	Coffee	Bleaching	8.33 (±5.53)
		Polishing	3.79 (±1.02)
		Total	6.06 (±4.55)
	Total	Bleaching	5.61 (±4.48)
		Polishing	3.21 (±1.68)
		Total	4.41 (±3.58)

## Data Availability

Data sharing is not applicable for this article.
